# *miRNA‐34c* Suppresses Osteosarcoma Progression In Vivo by Targeting Notch and E2F

**DOI:** 10.1002/jbm4.10623

**Published:** 2022-04-09

**Authors:** Yangjin Bae, Huan‐Chang Zeng, Yi‐Ting Chen, Shamika Ketkar, Elda Munivez, Zhiyin Yu, Francis H. Gannon, Brendan H. Lee

**Affiliations:** ^1^ Department of Molecular and Human Genetics Baylor College of Medicine Houston TX USA; ^2^ Integrative Molecular and Biomedical Sciences Program Baylor College of Medicine Houston TX USA; ^3^ Department of Pathology and Immunology Baylor College of Medicine Houston TX USA

**Keywords:** E2F5, miRNA‐34c, Notch, Osteosarcoma, Tumor suppressor

## Abstract

The expression of microRNAs (miRNAs) is dysregulated in many types of cancers including osteosarcoma (OS) due to genetic and epigenetic alterations. Among these, *miR‐34c*, an effector of tumor suppressor P53 and an upstream negative regulator of Notch signaling in osteoblast differentiation, is dysregulated in OS. Here, we demonstrated a tumor suppressive role of *miR‐34c* in OS progression using in vitro assays and *in vivo* genetic mouse models. We found that *miR‐34c* inhibits the proliferation and the invasion of metastatic OS cells, which resulted in reduction of the tumor burden and increased overall survival in an orthotopic xenograft model. Moreover, the osteoblast‐specific overexpression of *miR‐34c* increased survival in the osteoblast specific p53 mutant OS mouse model. We found that *miR‐34c* regulates the transcription of several genes in Notch signaling (*NOTCH1*, *JAG1*, and *HEY2*) and in p53‐mediated cell cycle and apoptosis (*CCNE2*, *E2F5*, *E2F2*, and *HDAC1*). More interestingly, we found that the metastatic‐free survival probability was increased among a patient cohort from Therapeutically Applicable Research to Generate Effective Treatments (TARGET) OS, which has lower expression of direct targets of *miR‐34c* that was identified in our transcriptome analysis, such as *E2F5* and *NOTCH1*. In conclusion, we demonstrate that *miR‐34c* is a tumor suppressive miRNA in OS progression *in vivo*. In addition, we highlight the therapeutic potential of targeting *miR‐34c* in OS. © 2022 The Authors. *JBMR Plus* published by Wiley Periodicals LLC on behalf of American Society for Bone and Mineral Research.

## Introduction

Osteosarcoma (OS) is the most common primary malignant bone cancer, comprising approximately 20% of all bone tumors and about 5% of pediatric tumors. It is the third most common type of cancer among children after leukemia and lymphoma. It has a bimodal distribution that peaks at under age 15 years and after age 65 years.^(^ ^1^, ^2^ ^)^ OS metastasizes to the lungs and has a high relapse rate. Five‐year survival for patients with localized disease approaches 70%, but patients with pulmonary metastasis experience poor survival rate of 19% to 30%.^(^ ^3^ ^)^ The genetic causes of OS are extensive but high penetrance mutations have been found in familial cancer syndromes associated with *RB1*, *TP53*, *RECQL4*, and *WRN*.^(^ ^4^ ^)^ In mice, the loss of p53 or Rb1 in osteoblastic lineages led to the spontaneous OS.^(^ ^5^, ^6^, ^7^ ^)^ Moreover, our group showed that gain of Notch1 in osteoblasts was also sufficient to initiate spontaneous OS.^(^ ^8^ ^)^ We and others have shown that Notch1 gain of function in early osteoblastic lineage cells leads to osteosclerosis due to proliferation of immature osteoblasts and inhibition of their differentiation into mature osteoblasts.^(^ ^9^, ^10^, ^11^, ^12^ ^)^ Long term, this also led to the development of osteogenic sarcomas that mimicked human OS in terms of distribution and molecular signatures.^(^ ^8^ ^)^ As Notch signaling is also upregulated in human OS samples, our transcriptional profiling of OS from p53 mutant mice also exhibited activation of Notch signaling.^(^ ^13^ ^)^

Tumor suppressor p53 protein not only regulates the transcription of coding genes but also regulates the transcription of noncoding RNAs such as microRNAs (miRNAs). Among these, the evolutionally conserved miR‐34 family (*miR‐34a*, *b*, and *c*) has been reported as a tumor suppressor in response to DNA damage and oncogenic stress and induces apoptosis and cell‐cycle arrest.^(^ ^14^, ^15^ ^)^ Interestingly, downregulation of *miR‐34* expression was seen in many types of human cancers such as colon, glioma, and non‐small cell lung cancers.^(^ ^16^, ^17^, ^18^ ^)^ In addition, it has been shown that genetic and epigenetic alterations in OS lead to decreased *miR‐34* expression level.^(^ ^19^, ^20^, ^21^ ^)^ These findings suggest an important role of miR‐34 as a downstream factor of p53 and a potential tumor suppressor miRNA in OS, although this has not been tested *in vivo*.

In our previous study, we found that *miR‐34c* was one of the significantly upregulated miRNAs during BMP‐2–mediated C2C12 osteoblast differentiation. More interestingly, we and others elucidated its essential role in bone homeostasis using a *Col1a1 2.3kb‐miR‐34c* transgenic mouse model.^(^ ^22^, ^23^ ^)^ We showed that the gain of *miR‐34c* in osteoblasts regulates osteoclastogenesis via non‐cell autonomous manner, which phenocopies the osteoblast‐specific loss‐of‐function Notch1 mouse model.^(^ ^22^ ^)^ Mechanistically, we confirmed that *miR‐34c* directly targets the 3′ untranslated region (3′UTR) of multiple components of Notch signaling (*Notch1*, *Notch2*, and *Jag1*) to regulate bone homeostasis in the transgenic mouse model.^(^ ^22^ ^)^ Therefore, we hypothesized that the p53 downstream effector *miR‐34c* targets Notch signaling and other pathways/targets to inhibit OS tumorigenesis.

In this study, we demonstrate the tumor suppressive role of *miR‐34c* by using an orthotopic xenograft model and a genetic mouse model of the osteoblast‐specific *miR‐34c* gain‐of‐function on a spontaneous OS osteoblast‐specific p53 mutant background. Mechanistically, we showed that *miR‐34c* regulates Notch signaling and other cancer related pathways by targeting multiple genes (*CCNE2*, *E2F2*, *E2F5*, and *HDAC1*). Moreover, we found that the metastasis‐free survival was increased among the Therapeutically Applicable Research to Generate Effective Treatments (TARGET) OS cohort, which had lower expression of *E2F5* and *NOTCH1*. Overall, our findings provide genetic support for a tumor suppressive function of *miR‐34c* in mouse and human OS. In addition, our study highlights the therapeutic potential for targeting *miR‐34c* in OS progression.

## Materials and Methods

### Animals

*Col1a1 rat 2.3kb Cre*; *p53* ^*f/f*^ mice were kindly provided by Dr. Jason Yustein (Baylor College of Medicine, Houston, TX, USA).^(^ ^7^ ^)^ In brief, *Col1a1 rat 2.3kb Cre*; *p53* ^*f/f*^ is generated by crossing *Col1a1 2.3kb* rat promoter driving a *Cre* transgene in osteoblasts with *p53* ^*f/f*^ mice (C57BL/6 background).^(^ ^24^ ^)^ To test the tumor suppressive role of *miR‐34c* in OS, we crossed *Col1a1 rat 2.3kb Cre*; *p53* ^*f/f*^ mice with *Col1a1 2.3kb‐miR‐34c* transgenic mice (FVB/N background).^(^ ^22^ ^)^ The established genetic mouse line of *Col1a1 rat 2.3kb Cre*; *p53* ^*f/f*^; *Col1a1 2.3kb‐miR‐34c* is a mixed background of FVBN and C57BL/6. All research performed with these mice was conducted in compliance with the Baylor Animal Protocol Committee (Baylor College of Medicine Animal Protocol AN5136). All animals had comprehensive necropsies upon the time of euthanasia with complete dissection of tumor along with other organs including the lungs, liver, and other bones. Portion of tumor and lungs were processed for the pathology for the histological analysis and lung metastasis.

### Cell lines and cell culture

The human osteosarcoma cell lines—143B, SJSA1, SAOS2, U2OS, and MG63 (CLS Cat# 300441)—were purchased from ATCC (American Type Culture Collection, Manassas, VA, USA). 143B and MG63 cells were maintained in minimum essential medium (MEM) (Gibco, Grand Island, NY, USA). SAOS2 and U2OS cells were maintained in McCoy's 5A medium. SJSA1 cells were maintained in RPMI‐1640 medium. All media were supplemented with 10% fetal bovine serum (FBS) (Gibco) and 1% penicillin and streptomycin (Gibco).

Stable clonal cell lines of LG34C or LGS were established by sequentially infecting lentivirus expressing luciferase followed by lentivirus expressing either *miR‐34c*‐GFP or SCR‐GFP in 143B parental cells (Fig. S2A). In brief, the pLenti PGK V5‐LUC Neo (Addgene plasmid # 21471) was obtained from Addgene (Cambridge, MA, USA).^(^ ^25^, ^26^ ^)^ The pGIPZ was purchased from Open Biosystems (Huntsville, AL, USA; vector # RHS4346). A 370‐bp fragment of genomic DNA containing miRNA *miR‐34c* was cloned into the *Xho I* and *Mlu I* restriction sites of the pLKO.1 vector (Addgene plasmid #52920) to express *miR‐34c*.^(^ ^27^ ^)^ The nonsilencing control of the pGIPZ vector served as the scramble control. Neomycin (125 mg/mL) and puromycin (1 μg/mL) were treated for the bulk selection of stable cells. Further, a single clone from each cell line was assessed for luciferase intensity and expression level of *miR‐34c* and *NOTCH1*. Based on these criteria, we selected and established a stable single clonal cell line of LG34C or LGS.

### Invasion assay

The invasion assays were conducted in 24‐well plates with permeable supports on 8‐μm Pore Polycarbonate Membrane (Corning Transwell 3422; Corning, Inc., Corning, NY, USA) for 24 and 48 hours.^(^ ^28^ ^)^ Eight micrograms/microliter (8 μg/μL) of Matrigel matrix (Sigma‐Aldrich, St. Louis, MO, USA; E1270) was coated on the permeable supports (upper chambers) and incubated at 37°C for 1 hour. Cell suspensions prepared in serum‐free media were seeded onto the upper chamber (1 × 10^4^ cells/chamber) and culture media containing 10% FBS was loaded in the lower chamber. After the incubation, invaded cells in the lower chamber were washed with PBS and stained with 0.05% crystal violet for 1 hour. The invaded cells were captured under the microscope and counted in five random fields in triplicate.

### Cell proliferation assay

Cells were plated in 96‐well plates (2500 cells/well) and grown for 48 and 72 hours. 3‐(4,5‐Dimethylthiazol‐2yl)‐2,5‐diphenyltetrazolium bromide (MTT) reagent (MTT Cell Proliferation Assay; ATCC; ATCC 30‐1010K) were added into each well. Cells were then incubated with MTT reagent for 4 hours. After 4 hours of incubation, the media were removed and 100 μL of DMSO were added to resolve purple precipitate. After 30 minutes under the room temperature, the absorbance in each well was measured at 570 nm in a microtiter plate reader. Medium only wells were processed with the same procedure for the background reading. The average values of absorbance readings were subtracted with the average value of the blank.

### Gene expression analysis by qRT‐PCR

Total RNA was extracted with TRIzol® (Thermo Fisher Scientific, Waltham, MA, USA), and cDNA was synthesized using Superscript III First‐Strand RT‐PCR kit (Thermo Fisher Scientific). RNA expression was analyzed by qPCR with SYBR Green I reagent (Roche Diagnostics, Mannheim, Germany). *ACTB* was used as reference gene for the normalization. For miRNA qRT‐PCR, TaqMan MicroRNA Assay (Applied Biosystems, Foster City, CA, USA) were used to quantify the expression of mature *miR‐34c* (Assay ID:000428). TaqMan Universal PCR Master Mix was used for amplification and detection. Sno202 RNA was used as reference gene for the normalization.

### Overexpression of miR‐34c

143B cells were plated into a six‐well plate and transfected with 100nM of *miR‐34c* mimic or negative miRIDIAN mimic using Lipofectamine 2000 (Thermo Fisher Scientific) according to the manufacturer's instructions. Cells were harvested 48 hours after transfection for targets' mRNA analysis.

### RNA sequencing and data analysis

Total RNA was extracted from 143B stable cell lines (LGS and LG34C) using TRIzol® (Thermo Fisher Scientific). Extracted RNA was then further purified by lithium chloride (Thermo Fisher Scientific) precipitation. RNA quality and quantity were measured by Bioanalyzer (Agilent Technologies, Santa Clara, CA, USA). For RNA sequencing (RNA‐Seq), 250 ng of total RNA was used for QuantSeq 3′ mRNA‐seq library preparation (Lexogen, Wien, Austria) according to manufacturer's instructions. The alignment was performed using HISAT2 (https://daehwankimlab.github.io/hisat2/) through Genialis (Boston, MA, USA; https://www.genialis.com) with hg19‐ERCC (human genome reference 19 build with ERCC spike‐in sequences) as reference. Normalization, differential expression, hierarchical clustering, and Gene Ontology (GO) analysis were then performed using EnrichR (https://maayanlab.cloud/Enrichr/) provided from Genealis.

### Human data analysis using TARGET OS

The gene expression profiles and clinical information data of the OS patients were obtained from the TARGET (https://ocg.cancer.gov/programs/target) initiative, phs000468. The RNA‐Seq data of 87 osteosarcoma patient samples were obtained from TARGET data (https://portal.gdc.cancer.gov/projects). The *Z*‐scores of the gene expression (transcripts per million [TPM]) of TARGET OS patients were binned into tertiles. Survival analysis of the top and bottom tertiles and metastasis‐free survival time was performed with the Kaplan‐Meier method using function *survfit* from R package *survival* (R Foundation for Statistical Computing, Vienna, Austria; https://www.r-project.org/).

### Xenograft study

143B‐LG34C or 143B‐LGS cells were harvested and resuspended in cold PBS. For each injection, 10,000 cells were mixed with 1:1 ratio with cell suspension and Matrigel, and total 50 μL was intratibially injected into athymic nude mice (Hsd;Athymic Nude‐*Foxn1* ^*nu*^) (10 mice/group).^(^ ^7^, ^29^, ^30^ ^)^ Tumor growth were monitored by the intraperitoneal (i.p.) injection of D‐luciferin by *in vivo* whole animal bioluminescence imaging (IVIS Xenogen; Xenogen, Alameda, CA, USA). Animals were imaged weekly to monitor tumor growth and lung metastasis.

### Statistical analysis

To determine the statistical significance among groups, Student's *t* test or analysis of variance (ANOVA) test were performed followed by multiple comparison test (*p < 0.05; **p < 0.01; ***p < 0.005; ****p < 0.0001; NS = not significant). Data are presented as mean ± standard deviation (SD) or mean ± standard error of the mean (SEM). Kaplan‐Meier calculations were used to show the overall survival time and the results were compared by a log‐rank (Mantel‐Cox) test. Fisher's exact test was used for the prevalence of lung metastasis in the genetic mouse models.

## Results

### miR‐34c expression is reduced in the metastatic human osteosarcoma cell lines while Notch signaling components are activated in complementary fashion

**Fig. 1:**
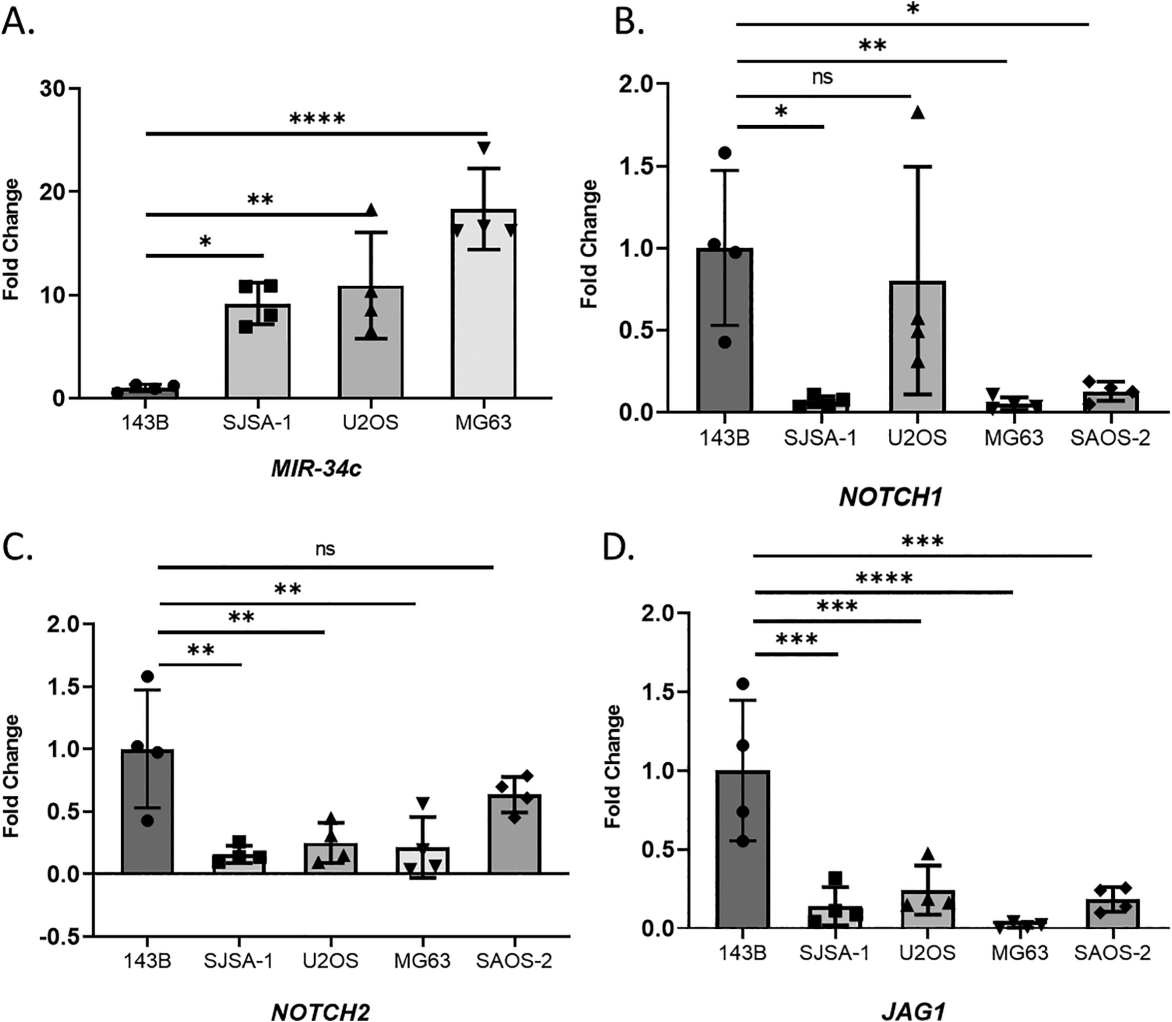
Expression of *MIR‐34c* and Notch signaling components in hOS cell lines. qRT‐PCR analysis of *MIR‐34c* (*A*), *NOTCH1* (*B*), *NOTCH2* (*C*), and *JAG1* (*D*) expression in hOS cell line (143B, SJSA‐1, U2OS, MG63, and SAOS‐2). Values are mean ± SD, *n* = 4. *p < 0.05; **p < 0.01; ***p < 0.001; ****p < 0.0001 (one‐way ANOVA and Sidak test for multiple comparison with 143B). hOS = human osteosarcoma.

The expression of the miR‐34 family is dysregulated in many cancers due to genetic alterations and epigenetic modifications.^(^ ^31^, ^32^ ^)^ Therefore, we first accessed the expression level of *MIR‐34c* in human OS cell lines. *MIR‐34c* expression was significantly reduced in 143B cells, a highly metastatic human OS cell line, compared to other OS cell lines such as SJSA1, U2OS, MG63, and SAOS‐2 (Fig. 1 *A*, Fig. S1).^(^ ^33^, ^34^, ^35^ ^)^ Consistent with our finding, others have reported that *MIR‐34c* expression in OS cell lines was decreased compared to human fetal osteoblast cells (hFOBs).^(^ ^32^ ^)^ In addition, we found Notch signaling components including *NOTCH1*, *NOTCH2*, and *JAG1* expression were significantly elevated in high metastatic potential 143B cells compared to other human OS cell lines (Fig. 1*B*–*D*). This expression pattern demonstrates an inverse correlation of *miR‐34c* versus Notch expression in the metastatic human OS cell line. This result is consistent with our previous report demonstrating *miR‐34c*'s direct inhibition of Notch signaling during physiological osteoblast differentiation.^(^ ^22^ ^)^ Given that miR‐34c is a downstream effector of p53 and an upstream negative regulator of Notch signaling, we hypothesized that *miR‐34c* plays a tumor suppressive role in OS progression.

### miR‐34c inhibits proliferation and invasiveness of metastatic human OS cell line

**Fig. 2:**
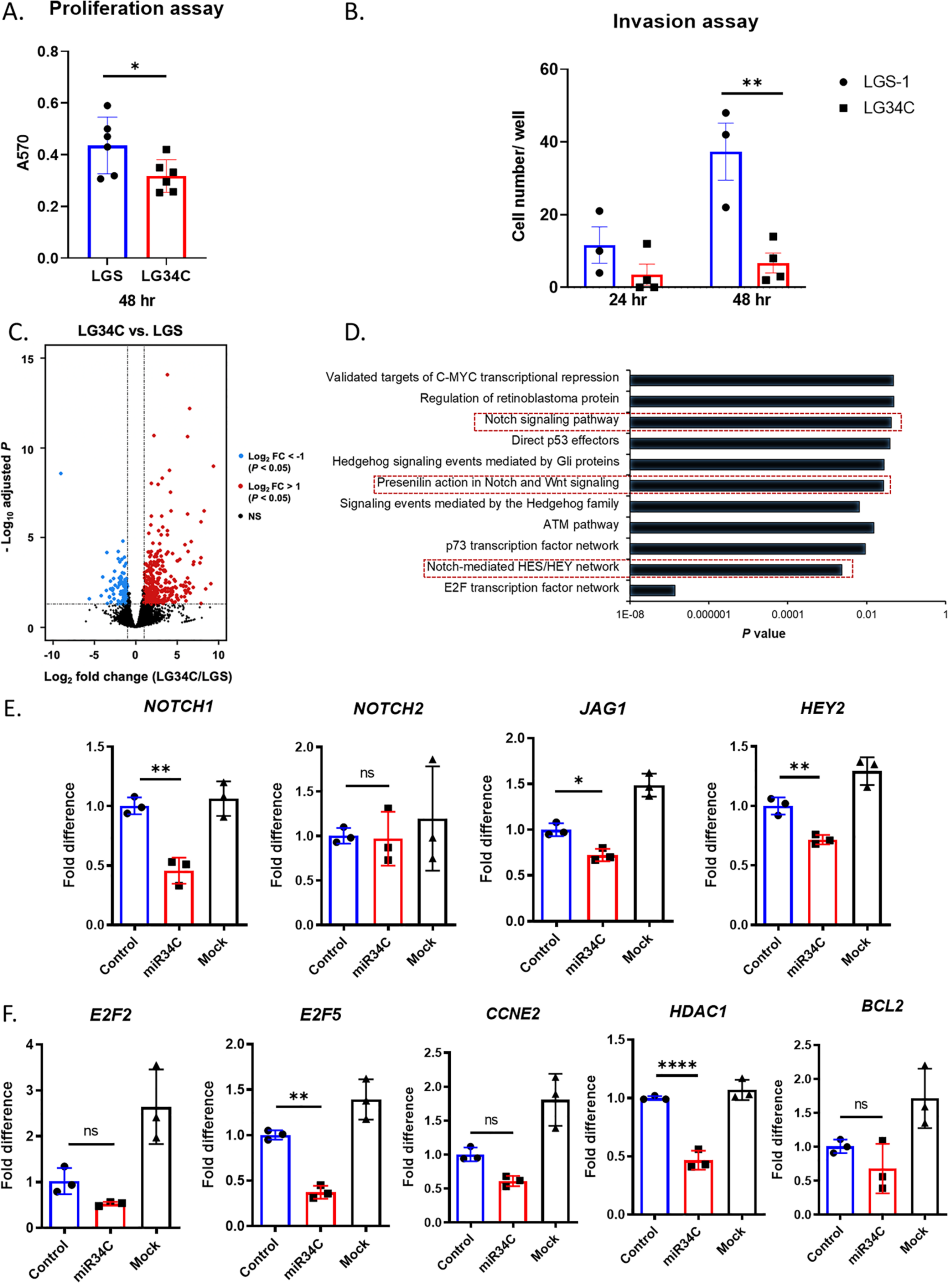
Gain‐of‐function *MIR‐34c* in 143B decreases cell proliferation and invasion. (*A*) Decreased cell proliferation in *MIR‐34c*–expressing 143B cells by MTT assay at 48 hours. Values are mean ± SD, *n* = 6. *p < 0.05 (Student's *t* test). (*B*) Decreased invasion in *MIR‐34c*–expressing cells at 24 and 48 hours. Values are mean ± SD, *n* = 3. **p < 0.01(Student's *t* test). LGS (Luciferase‐GFP‐Scramble control stably expressing in 143B) and LG34C (Luciferase‐GFP‐*miR‐34c* stably expressing in 143B). (*C*) Volcano plot of differentially expressed genes between LG34C and LGS with a log_2_ fold change +/−1 and *p* value cutoff 0.05. Red dot indicates upregulated genes and blue dot indicates downregulated genes. (*D*) Pathway affected in LG34C versus LGS within downregulated genes. (*E*) qRT‐PCR analysis of Notch signaling pathway genes and (*F*) E2F transcription networks genes from 143B cells transfected with *miR‐34c* mimic (miR34C) or control mimic (Control). Values are mean ± SD, *n* = 3.*p < 0.05; **p < 0.01; ***p < 0.001; ****p < 0.0001 (one‐way ANOVA and Sidak test for multiple comparison with control versus miR34C group). FC = fold change.

To test the tumor suppressive potential of *miR‐34c* in OS, we generated a series of clonal cell lines of overexpressing *miR‐34c* (LG34C: Luciferase‐GFP‐*miR‐34c*) and their scramble control (LGS: Luciferase‐GFP‐Scramble) from 143B cells. The construct contains luciferase to enable live cell imaging, the quantitative analysis of tumor growth, and lung metastasis over time using bioluminescence imaging (Fig. S2A). After establishing eight clonal cell lines from each LG34C and LGS control, we compared the luciferase activity by % luminescence and further confirmed the overexpression of *miR‐34c* (Fig. S2B) and the inverse expression of *NOTCH1* (Fig. S2C) in LG34C lines versus LGS control lines. The cellular effects of the gain‐of‐*miR‐34c* in 143B cells were then assessed. Overexpression of *miR‐34c* (LG34C) resulted in decreased cell proliferation at 48 hours (Fig. 2*A*), but there was no significant difference at 72 hours (Fig. S2D). In addition, overexpression of *miR‐34c* reduced invasion at 24 and 48 hours compared to the LGS (Fig. 2*B*).These cellular phenotypes suggest that *miR‐34c* can affect the tumor burden and the metastatic property in OS progression.

### miR‐34c regulates Notch signaling including cancer‐related pathways in OS tumorigenesis

To elucidate the underlying molecular mechanisms of tumor suppressive role of *miR‐34c* in OS progression, we performed 3′‐mRNA sequencing using three biological replicates of each clonal cell line (Control group: LGS1, LGS4, and LGS8; miR‐34c group: LG34C3, LG34C5, and LG34C7). We obtained significantly altered expression of 110 downregulated genes (*p* < 0.05 and log_2_ fold change < −1) and 418 upregulated genes (*p* < 0.05 and log_2_ fold change >1) (Fig. 2*C*). Subsequently, we focused on the differentially downregulated genes to identify putative direct targets of *miR‐34c*. Among downregulated genes, we found that 22 genes were candidate direct targets of *miR‐34c* based on the TargetScan analysis (Table S1). Among affected pathways based on GO analysis of these downregulated genes, the E2F transcription factor network seemed most significantly affected (*p* = 1.38E−07) (Fig. 2*D*). In addition, Notch signaling (Notch‐mediated HES/HEY network, Presenilin action in Notch and Wnt signaling, and Notch signaling pathway) was found as a recurrently affected pathway (Fig. 2*D*). Several targets representing these pathways are grouped in Fig. S2E. We confirmed these targets independently by overexpressing *miR‐34c* mimic in 143B cells. *NOTCH1*, *JAG1*, and *HEY2* transcripts were significantly downregulated (Fig. 2*E*). This is consistent with our previous transcriptome analysis performed with calvarial tissue from mice expressing *miR‐34c* from the *Type I Collagen* promoter (*Col1a1 2.3kb‐miR‐34c*), in which many cancer‐associated pathways including Notch signaling was dysregulated.^(^ ^22^ ^)^ We also found that *Direct p53 effectors*, *E2F transcription factor network*, and *Regulation of RB protein* were differentially regulated, and genes associated in these pathways—*E2F2*, *E2F5*, *HDAC1*, *BCL2*, and *CCNE2*—were downregulated (Fig. 2*F*). Among these, *E2F5* and *HDAC1* showed significantly suppressed expression (Fig. 2*F*). Overall, the transcriptome analysis and target analysis in OS cell lines showed that *miR‐34c* regulates multiple cancer‐related pathways and molecular regulators including the Notch signaling pathway.

### miR‐34c overexpression reduces tumor burden in vivo in a xenograft model of OS

**Fig. 3:**
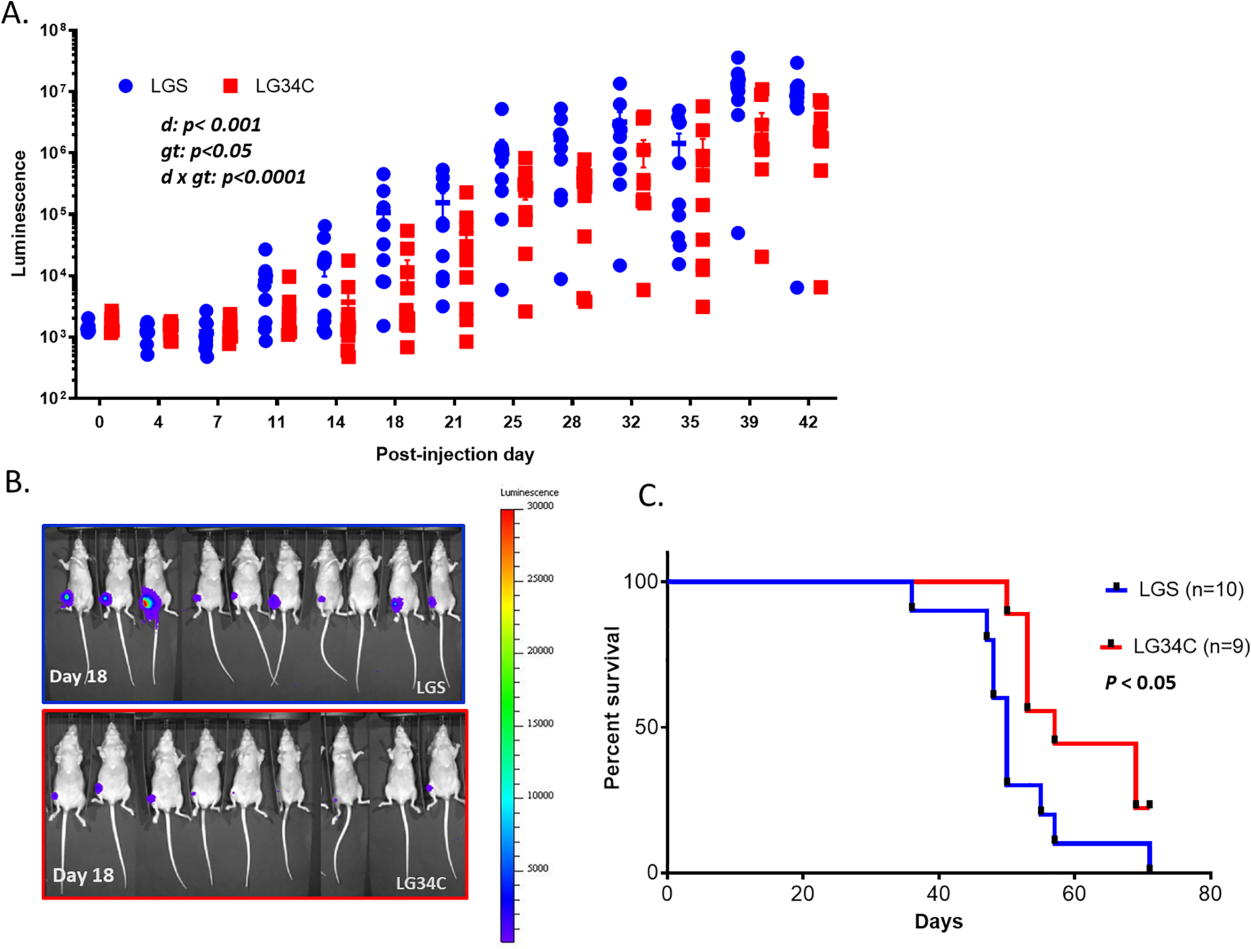
Gain of *miR‐34c* significantly suppressed tumor growth *in vivo* and increased survival in xenograft model. (*A*) Tumor burden was quantified by luminescence intensity measured by *in vivo* whole animal bioluminescence imaging up to day 42 postinjection. *n* = 9. *p < 0.05; ***p < 0.001; ****p < 0.0001 (two‐way ANOVA). Sidak multiple comparison was performed. (*B*) *In vivo* live bioluminescence imaging of tumors from each LGS and LG34C engrafted mice at day 18 postinjection. (*C*) Kaplan‐Meier survival analysis showed increased percent survival of LG34C compared to LGS. *p* < 0.05 (log‐rank test). d = day; gt = genotype.

To determine the tumor suppressive function of *miR‐34c in vivo*, we performed a xenograft study by intratibial (IT) injection of *miR‐34c* overexpressing LG34C or LGS control cell lines in immune‐deficient mice (Fig. S3A). We monitored tumor growth and lung metastasis by detection of luciferase expression by bioluminescence imaging (Fig. S2A). Consistent with a decreased proliferation observed with overexpression of miR‐34c in LG34C cells (Fig. 2*A*), we found reduced tumor burden in LG34C compared to LGS engrafted immune‐deficient mice (Fig. 3*A*,*B*). More interestingly, we found the prolonged survival in the gain‐of‐function *miR‐34c* (LG34C) versus control (LGS) groups (Fig. 3*C*). Because we previously found a significant impact on the invasiveness of LG34C cells (Fig. 2*B*), we also accessed the pulmonary metastasis *in vivo*, which is a primary cause of death of OS patients. However, there was no difference in the burden of lung metastasis based on the level of luminescence from the lung samples at the termination in this aggressive immune‐deficient model (Fig. S3B). Overall, these collective results revealed that *miR‐34c* is a tumor suppressor in OS by inhibiting cell proliferation and invasiveness in vitro and decreasing tumor burden, thereby increasing overall survival *in vivo*. However, we did not observe any effect of *miR‐34c* on the pulmonary metastasis in our xenograft study.

### Gain of miR‐34c in osteoblasts improves survival of a spontaneous genetic osteosarcoma mouse model (*Col1a1 rat 2.3kb Cre*; *p53* ^*f/f*^)

**Fig. 4:**
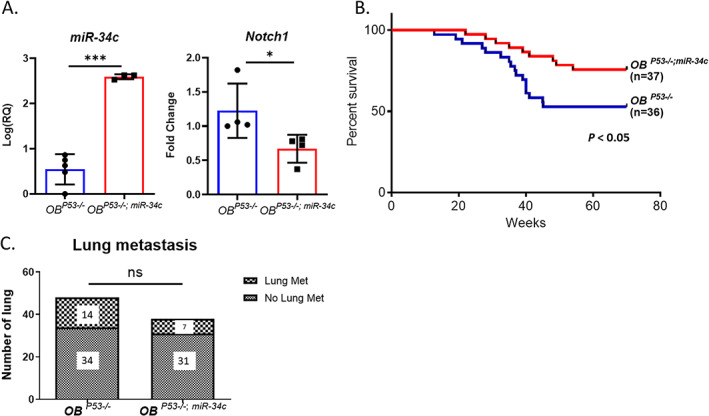
Genetic rescue by the osteoblast specific gain of *miR‐34c* in *Col1a1 rat 2.3kb Cre*; *p53* ^*f/f*^. (*A*) qRT‐PCR analysis of *miR‐34c* and *Notch1* from the calvarial tissue of each mouse line. Values are mean ± SD, *n* = 5. *p < 0.05, ***p < 0.001 (Student's *t* test). (*B*) Kaplan‐Meier survival analysis showed increased percent survival of *OB* ^*p53−/−*; *miR*−*34c*^ compared to *OB* ^*p53−/−*^. *p* < 0.05 (log‐rank test). (*C*) No significant difference was found in the incidence of lung metastasis (Fisher's exact test). ns = not significant.

Deletion of TP53, or RB1 in the osteoblastic lineage cells led to the spontaneous osteosarcoma in the genetic mouse models.^(^ ^5^, ^6^, ^7^ ^)^ Moreover, human genetics show that the mutation or loss of these genes predispose to OS.^(^ ^4^ ^)^ Interestingly, the transcriptional analysis of OS from p53 mutant mice also showed significantly increased many components of Notch signaling pathway.^(^ ^13^ ^)^ In addition, we have demonstrated that the gain‐of‐function Notch1 in mature osteoblasts is sufficient to cause spontaneous OS in a genetically engineered mouse model.^(^ ^8^ ^)^ More important, we found elevated Notch signaling in primary human OS samples and even higher in metastatic OS samples.^(^ ^13^ ^)^ To demonstrate the *in vivo* tumor suppressive role of *miR‐34c* in OS, we over expressed *miR‐34c* in the osteoblasts by intercrossing *Col1a1 2.3kb‐miR‐34c* transgenic mice with *p53* deletion in osteoblasts (*Col1a1 rat 2.3kb Cre*; *p53* ^*f/f*^: refer as *OB* ^*p53−/−*^).^(^ ^7^, ^22^, ^24^ ^)^ We confirmed that *miR‐34c* transcript level was elevated in the calvarial tissue of gain‐of‐function *miR‐34c* transgenic mice in the absence of *p53* in the mature osteoblasts (*Col1a1 rat 2.3kb Cre*; *p53* ^*f/f*^; *Col1a1 2.3kb‐miR‐34c*: refer as *OB* ^*p53−/−*; *miR*−*34c*^) by qRT‐PCR (Fig. 4*A*). As expected from gain‐of‐function *miR‐34c*, *Notch1* expression was decreased in the calvarial tissue of *OB* ^*p53−/−*; *miR*−*34c*^ (Fig. 4*A*). We found that *OB* ^*p53−/−*; *miR*−*34c*^ mice showed increased survival rate compared to *OB* ^*p53−/−*^, indicating that *miR‐34c* functions as a tumor suppressor in OS (Fig. 4*B*). We also found a trend toward lower incidence of lung metastasis in *OB* ^*p53−/−*; *miR*−*34c*^ mice compared to *OB* ^*p53−/−*^ alone (Fig. 4*C*). Overall, our genetic model of gain‐of‐function *miR‐34c* in OS mouse model along with the orthotopic xenograft study confirms tumor suppressive miRNA in OS progression by increasing survival rate.

### Increased expression of miR‐34c targets in human OS patients associated with poor metastasis‐free survival

**Fig. 5:**
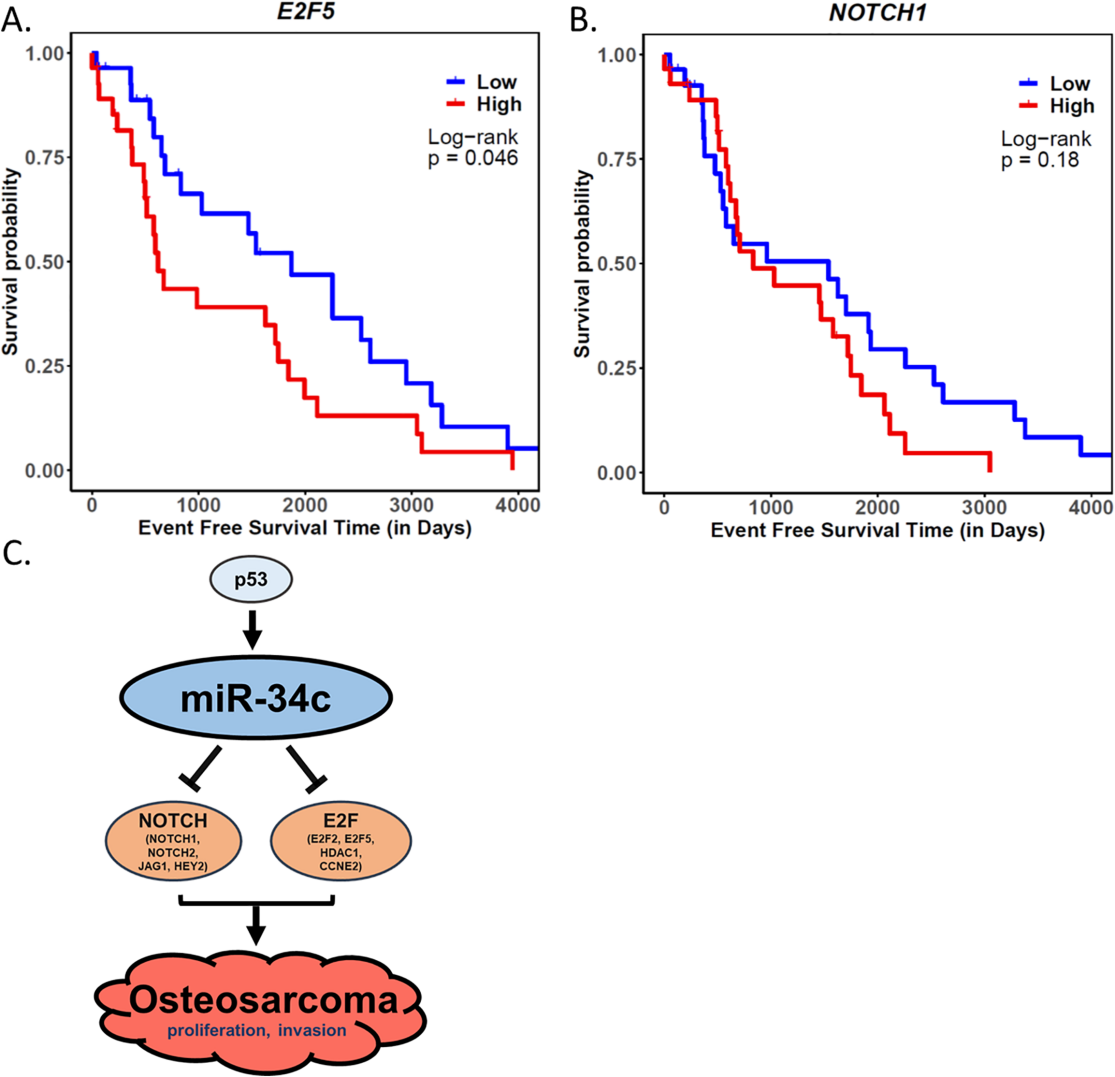
Analysis of *miR‐34c* target expression and metastasis‐free survival of the TARGET‐OS patient cohort. (*A*) *E2F5* and (*B*) *NOTCH1* expression versus metastasis‐free survival of TARGET‐OS patient cohort (*n* = 87) by Kaplan‐Meier survival analysis (log‐rank test). (*C*) Schematic summary of tumor suppressive role of miR‐34c by targeting Notch and E2F in OS progression.

In order to assess the clinical relevance of targets identified from the transcriptome analysis of stably overexpressing *miR‐34c* 143B cells, we employed the TARGET OS cohort (*n* = 87) and investigated the correlation between the expression level of these targets and the metastasis‐free survival of OS patients. Kaplan‐Meier survival analysis for these target genes, binned as tertiles, shows the metastasis‐free survival probability of a TARGET OS patient in Fig. 5. Interestingly, we found higher survival rate of OS patients who have lower expression of *E2F5* and *NOTCH1* (Fig. 5*A*,*B*). This metastasis‐free survival analysis corroborates our findings of extended survival in *Col1a1 rat 2.3kb Cre*; *p53* ^*f/f*^; *Col1a1 2.3kb‐miR‐34c* (Fig. 4*B*) and the xenograft models of OS progression (Fig. 3*C*).

In conclusion, our study demonstrated the tumor suppressive role of *miR‐34c* in OS progression by targeting multiple pathways including Notch signaling and cell cycle regulators (Fig. 5*C*). This was associated with decreased proliferative and invasive properties of OS cells in vitro. Moreover, we showed a tumor suppressive function by the genetic interaction of osteoblast specific gain‐of‐function *miR‐34c* with p53 loss‐of‐function in a spontaneous OS mouse model. Finally, we identified clinical significance of *miR‐34c* targets by correlating the OS patient survival with a subset of these targets using the TARGET OS cohort. Our findings provide genetic evidence in support of the tumor suppressive function of the *miR‐34c* during OS progression and identify it as a potential therapeutic target for OS.

## Discussion

Dysregulation of miRNAs by genetic and epigenetic alterations has been widely implicated in tumorigenesis, which contributes to their potential roles as oncogenes and tumor suppressors.^(^ ^20^, ^21^ ^)^ Such alterations were also observed in OS compared to healthy bone.^(^ ^14^ ^)^ Among these, the miRNA‐34 family is subjected to genetic and epigenetic changes in OS. There is decreased expression of *miR‐34c* due to gene deletions and to DNA hypermethylation.^(^ ^19^ ^)^ The restoration of the miR‐34 family in OS cell lines partially induced cell‐cycle arrest, apoptosis, and delay proliferation and invasion, indicating a potential tumor suppressive role of the miR‐34 family. Interestingly, we also found that *miR‐34c* expression was drastically suppressed in a highly metastatic human osteosarcoma cell line (143B) compared to low‐metastatic potential cell lines (Fig. 1), and the overexpression of *miR‐34c* can suppress the proliferative and invasive properties of metastatic 143B cells. Previously, we and others demonstrated that miR‐34c is one of the osteogenic miRNAs that also plays important roles in bone homeostasis.^(^ ^22^, ^23^ ^)^ We further showed that miR‐34c regulates Notch signaling by directly targeting many of its components (*Notch1*, *Notch2*, and *Jag1*) in osteoblasts.^(^ ^22^ ^)^ Notch signaling is tightly regulated to maintain a balance of pools of progenitors versus differentiated cells during bone homeostasis. Not surprisingly, dysregulation of Notch signaling has also been implicated in various cancers including OS. We found Notch signaling components were highly upregulated in primary and metastatic tumors from the OS patients.^(^ ^13^ ^)^ In addition, these components were highly elevated in OS from *p53* ^*+/−*^ mice, suggesting the functional interaction of tumor suppressor p53 and Notch in OS progression. The definitive oncogenic role of Notch signaling in OS was further proven by spontaneous OS development in an osteoblast‐specific gain‐of‐function Notch mouse model. Moreover, there was a synergistic effect between p53 and Notch in accelerating OS progression.^(^ ^8^ ^)^ Here, we assessed whether *miR‐34c* as an upstream regulator during bone homeostasis may similarly serve as a tumor suppressor in the context of OS. *miR‐34c* appears to serve this dual role in bone homeostasis and in OS progression. Functionally, because miRNAs target many genes and pathways, *miR‐34c* is a prime candidate for enhancing tumor suppressive function in OS.

Concepcion and colleagues^(^ ^36^ ^)^ showed that complete deletion of the miR‐34 family did not compromise normal development and spontaneous tumorigenesis in mice. Furthermore, in contrast to p53‐dificient mice, miR‐34 family–deficient animals did not display any increased susceptibility compared to irradiation‐induced or c‐Myc–induced B cell lymphoma mice. Similarly, miR‐34a alone had little or no effect on tumor formation in mice.^(^ ^37^ ^)^ It accelerated initiation and formation of tumors only when these mice were bred with other mice harboring oncogenic variant such as *KrasG12D* (Kras‐driven lung cancer model) or *APC* (colon cancer mouse model).^(^ ^37^, ^38^ ^)^ Together, these loss‐of‐function mouse models suggest that the miR‐34 family is largely dispensable for p53 function and although not sufficient to initiate tumorigenesis, it may serve as an essential progression factor. To test the *in vivo* tumor suppressive role of *miR‐34c* in OS pathogenesis, we crossed transgenic mice overexpressing *miR‐34c* in osteoblasts (*Col1a1 2.3kb‐miR‐34c*) to mice deficient for p53 in osteoblasts (*Col1a1 rat 2.3kb Cre*; *p53* ^*f/f*^ or p53 cKO). Gain of *miR‐34c* in p53 cKO OS mice improved survival compared to p53 cKO OS mice, supporting the tumor suppressive role in OS progression (Fig. 4*B*). Because the miR‐34 family functions as a mediator of p53, our ectopic expression of *miR‐34c* in osteoblasts could partially compensate the loss of p53 function in OS. Consistent with this finding, our xenograft study showed the decreased tumor burden and increased survival from the mice engrafted via IT injection with 143B cells stably expressing *miR‐34c* (LG34C) versus scramble control (LGS) (Fig. 3). However, we did not observed any improvement in pulmonary metastasis in either the genetic mouse model (Fig. 4*C*) and xenograft studies (Fig. S3B) despite significant decrease of invasiveness in *miR‐34c* gain‐of‐function cells. The discrepancy may be explained partly by the difference of *in vivo* versus *in vitro* sensitivity to *miR‐34c* overexpression and the complex tumor microenvironment involving extracellular matrix, blood vessels, and host immune cells. Moreover, the aggressive nature of this orthotopic immune‐deficient model may not provide sufficient time to progression to demonstrate a protective effect of *miR‐34c* expression.

Mechanistically, Notch signaling and its cancer‐related pathway were differentially regulated in *miR‐34c* stably expressing 143B cells. This result is consistent with our previous study of the transgenic mouse model of overexpressing miR‐34c in osteoblastic cells (*Col1a1 2.3kb‐miR‐34c*).^(^ ^22^ ^)^ Many targets of *miR‐34c* found in our transcriptome analysis from 143B cells were shared among different pathways. Among E2F family proteins, *E2F5* was significantly downregulated in *miR‐34c* overexpressing 143B cells (Fig. 2*F*). These factors directly bind to cell‐cycle–associated factors and promote cell‐cycle progression.^(^ ^39^ ^)^ It is reported that *E2F5* was dysregulated in OS and shown to be downregulated by direct targeting of *miR‐154‐5p* in OS.^(^ ^40^ ^)^ *CCNE2*, involved in G0/G1 cell cycle regulation, was also downregulated in our transcriptome analysis, suggesting that inhibition of cell proliferation is one of the mechanisms of *miR‐34c*–mediated suppression in OS development. In addition, *HDAC1* was significantly downregulated in our transcriptome analysis. Treatment with panobinostat, a broad spectrum histone deacetylase (HDAC) inhibitor, showed decreased OS growth and lung metastasis in an orthotopic xenograft model using human OS cell lines.^(^ ^41^ ^)^ In this report, treatment with the selective inhibitor for HDAC1/2 compromised the growth of OS in vitro and *in vivo*. Moreover, Notch signaling is critical in maintenance of cell proliferation, because suppression of Notch signaling by *miR‐34c*, again effectively targets the cellular proliferative phenotype.

Finally, we examined the potential for some of *miR‐34c* targets to correlate with metastasis‐free survival of an OS patient cohort from the TARGET database. Notably, OS patients who have lower expression of *E2F5* among miR‐34c targets show higher metastasis‐free survival (Fig. 5*A*). Patients with lower expression of *NOTCH1* also tended to have a better prognosis (Fig. 5*B*). The expression level of other *miR‐34c* targets did not correlate with patient survival in the TARGET OS cohort. We also attempted to assess the metastasis‐free survival versus *miR‐34c* expression. However, the expression level of *miR‐34c* was mostly undetectable, so we were not able to perform such analysis. This reflects the mutations and epigenetic inactivation by DNA methylation of miR‐34b/c affecting the expression of miR‐34c and metastasis in OS.^(^ ^19^, ^21^ ^)^

Collectively, our study demonstrated that *miR‐34c* is not only a critical miRNA for the maintenance of bone homeostasis, but also a tumor suppressor in OS progression. It can inhibit the proliferation and invasion of tumor cells in vitro. It also decreased tumor burden in an orthotopic model of OS. Finally, *miR‐34c* overexpression in osteoblasts increased survival of the p53 cKO spontaneous OS model. Mechanistically, gain of *miR‐34c* regulates many targets from cancer‐related pathways and the Notch pathway. Among these, *E2F5* may significantly correlate with human OS progression because its elevated expression is associated with poorer survival of a patient cohort from TARGET OS. Our findings highlight the significance of *miR‐34c* targeting the multiple pathways/genes which could be candidates for OS therapy.

## Author Contributions

**Yangjin Bae:** Conceptualization; data curation; formal analysis; funding acquisition; writing – original draft. **Huan‐Chang Zeng:** Data curation; formal analysis; validation. **Yi‐Ting Chen:** Data curation; formal analysis; validation. **Shamika Ketkar:** Data curation; formal analysis; validation. **Elda Munivez:** Data curation. **Zhiyin Yu:** Data curation. **Francis Gannon:** Data curation; formal analysis; validation. **Brendan Lee:** Conceptualization; funding acquisition.

## Conflict of Interest

The authors declare no competing financial interests.

### Peer Review

The peer review history for this article is available at https://publons.com/publon/10.1002/jbm4.10623.

## Supporting information


**Fig. S1** Expression of *MIR‐34c* in SAOS‐2 human OS cell line. qRT‐PCR analysis of *MIR‐34c*. Values are mean ± SD, *n* = 4. ***, *p* < 0.001 (Student's *t* test).

**Fig. S2** Stable single clonal cell lines of LG34C and LGS in 143B cells. (A). Flowchart of generating lentivirus mediated stable expression of miR‐34c (LG34C) and scramble control (LGS) in 143B cells. (B). qRT‐PCR of *MIR‐34c* and (C) *NOTCH1* in single clonal cell lines from LGS and LG34C. (D). Cell proliferation in *MIR‐34c* expressing 143B cells by MTT assay at 72 hours. Values are mean ± SD, *n* = 6. ns = no significance (Student's *t* test). (E). Downregulated genes in affected pathways. Bolded genes in each pathway are directly targeted by miR‐34c based on the TargetScan analysis.

**Fig. S3** Effect of miR‐34c in tumor growth and lung metastasis *in vivo* using luciferase reporter in orthotopic xenograft model. (A). Intratibial injection was performed to deliver LGS or LG34C to immune incompetent mice. (B). Lung metastasis was monitored at the termination by ex vivo. No significant difference was found (Student's *t* test).

**Table S1** qRT‐PCR Primer Sets.

## Data Availability

The data support this study are available upon request to the corresponding author.
