# Supplementary figures and images for: miRNA‐34c Suppresses Osteosarcoma Progression In Vivo by Targeting Notch and E2F
Source: JBMR Plus. 2022 Apr 9;6(5):e10623. doi: 10.1002/jbm4.10623 (PMC9059472; doi:10.1002/jbm4.10623)

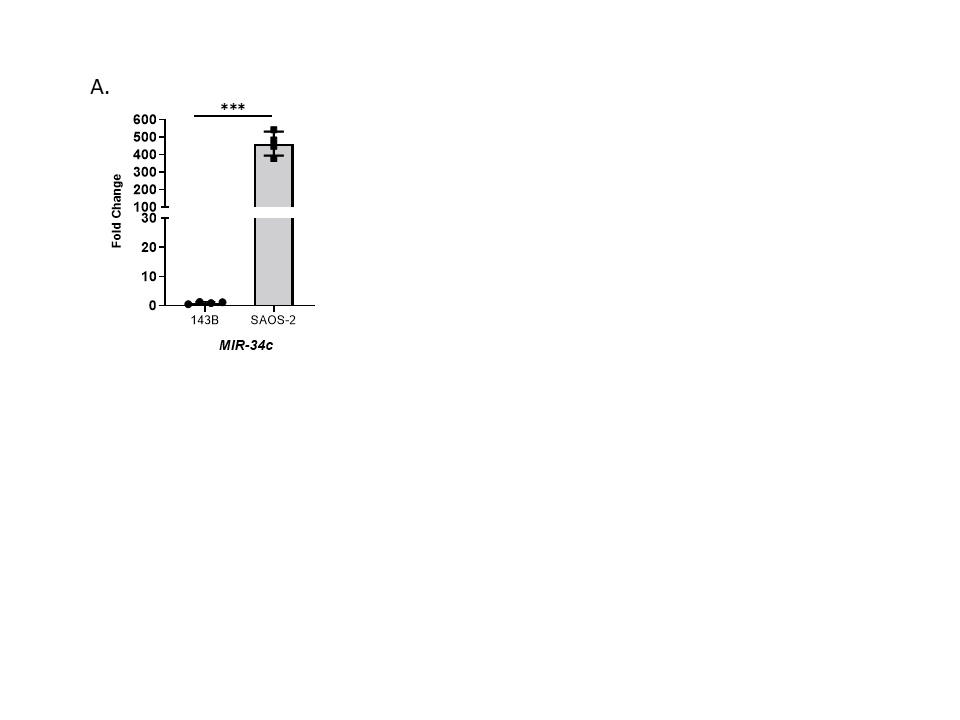

Supplement: Supplementary file 1 — Fig. S1 Expression of MIR‐34c in SAOS‐2 human OS cell line. qRT‐PCR analysis of MIR‐34c. Values are mean ± SD, n = 4. ***, p < 0.001 (Student's t test). [file JBM4-6-e10623-s004.tiff]

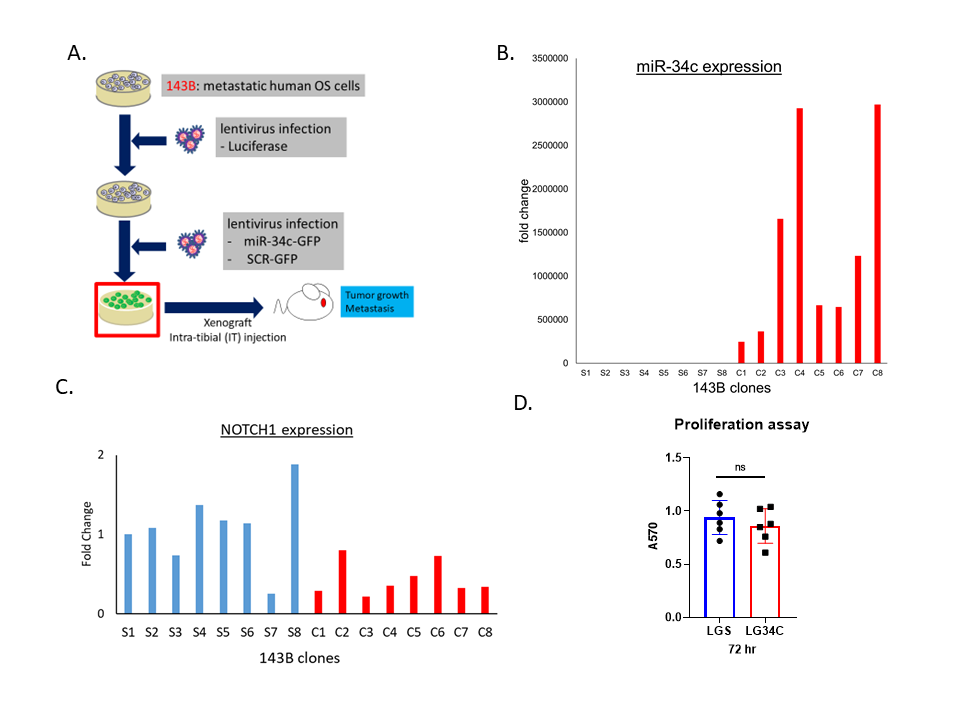

Supplement: Supplementary file 2 — Fig. S2 Stable single clonal cell lines of LG34C and LGS in 143B cells. (A). Flowchart of generating lentivirus mediated stable expression of miR‐34c (LG34C) and scramble control (LGS) in 143B cells. (B). qRT‐PCR of MIR‐34c and (C) NOTCH1 in single clonal cell lines from LGS and LG34C. (D). Cell proliferation in MIR‐34c expressing 143B cells by MTT assay at 72 hours. Values are mean ± SD, n = 6. ns = no significance (Student's t test). (E). Downregulated genes in affected pathways. Bolded genes in each pathway are directly targeted by miR‐34c based on the TargetScan analysis. [file JBM4-6-e10623-s002.zip › JBM4_10623_FIGURE S2.TIF]

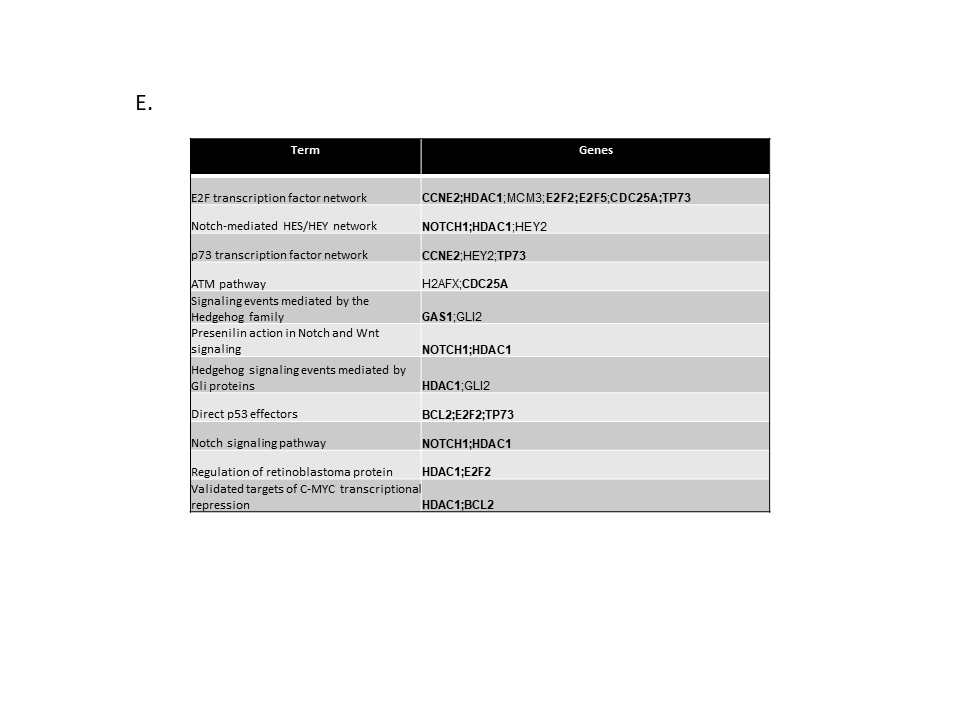

Supplement: Supplementary file 2 — Fig. S2 Stable single clonal cell lines of LG34C and LGS in 143B cells. (A). Flowchart of generating lentivirus mediated stable expression of miR‐34c (LG34C) and scramble control (LGS) in 143B cells. (B). qRT‐PCR of MIR‐34c and (C) NOTCH1 in single clonal cell lines from LGS and LG34C. (D). Cell proliferation in MIR‐34c expressing 143B cells by MTT assay at 72 hours. Values are mean ± SD, n = 6. ns = no significance (Student's t test). (E). Downregulated genes in affected pathways. Bolded genes in each pathway are directly targeted by miR‐34c based on the TargetScan analysis. [file JBM4-6-e10623-s002.zip › JBM4_10623_FIGURE S2-2.TIF]

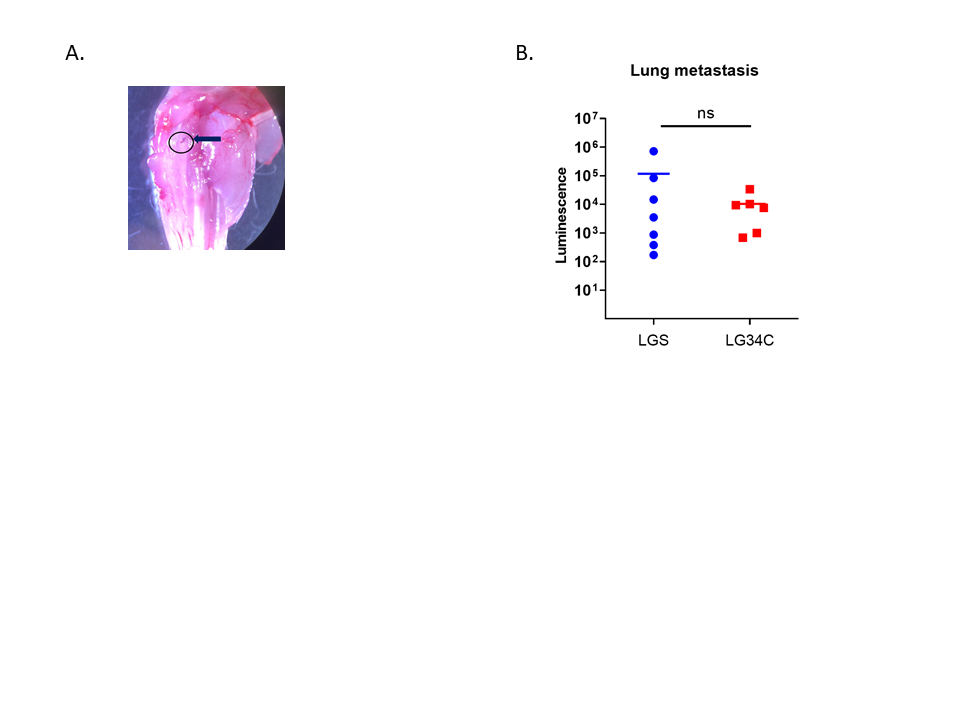

Supplement: Supplementary file 3 — Fig. S3 Effect of miR‐34c in tumor growth and lung metastasis in vivo using luciferase reporter in orthotopic xenograft model. (A). Intratibial injection was performed to deliver LGS or LG34C to immune incompetent mice. (B). Lung metastasis was monitored at the termination by ex vivo. No significant difference was found (Student's t test). [file JBM4-6-e10623-s003.tiff]
