# Supplementary material for: miRNA‐34c Suppresses Osteosarcoma Progression In Vivo by Targeting Notch and E2F
Source: JBMR Plus. 2022 Apr 9;6(5):e10623. doi: 10.1002/jbm4.10623 (PMC9059472; doi:10.1002/jbm4.10623)
Supplement: Supplementary file 4 — Table S1 qRT‐PCR Primer Sets. [file JBM4-6-e10623-s001.doc]

**Table S1.** qRT-PCR Primer sets.

| GENE | Primer | Sequence |
| --- | --- | --- |
| *NOTCH1* | Forward | CCATCGTCTACCTGGAGATTGACAAC |
|  | Reverse | CCCACGAAGAACAGAAGCACAAA |
| *NOTCH2* | Forward | CCTGTGCACCCTCACCTTGTGTCA |
|  | Reverse | TCTCATCGTGTTCTTTTCCATTCCA |
| *JAG1* | Forward | CCAGAGCTTAAACCGAATGGAGTACATC |
|  | Reverse | AGAACTACAAGCCCTCAGACTCTACCTA |
| *HEY2* | Forward | CGTGGGGAGCGAGAACAA |
|  | Reverse | CGGGTGGAGCGGATGA |
| *E2F2* | Forward | CGTCCCTGAGTTCCCAACC |
|  | Reverse | GCGAAGTGTCATACCGAGTCTT |
| *E2F5* | Forward | TCAGGACCTATCCATGTGCTGCTT |
|  | Reverse | TCAGAGACATGTTGCTCAGGCAGA |
| *CCNE2* | Forward | TAGACTGGCTTTTAGAGGT |
|  | Reverse | TTGGAGTTTAGGAGCATAGA |
| *HDAC1* | Forward | CTACTACGACGGGGATGTTGG |
|  | Reverse | GAGTCATGCGGATTCGGTGAG |
| *BCL2* | Forward | GCCCAGACAAATGTGGTTAC |
|  | Reverse | AATACAGGTCCTTCATACCCTTAGT |
| *ACTB* | Forward | GCCAACCGCGAGAAGATGACC |
|  | Reverse | CTCCTTAATGTCACGCACGATTTC |
